# The HR2 polymorphism N140I in the HIV-1 gp41 combined with the HR1 V38A mutation is associated with a less cytopathic phenotype

**DOI:** 10.1186/1742-4690-9-15

**Published:** 2012-02-14

**Authors:** Francesc Cunyat, Silvia Marfil, Elisabet García, Valentina Svicher, Nuria Pérez-Alvárez, Marta Curriu, Carlo Federico Perno, Bonaventura Clotet, Julià Blanco, Cecilia Cabrera

**Affiliations:** 1 IrsiCaixa-HIVACAT, Institut de Recerca en Ciències de la Salut Germans Trias i Pujol (IGTP), Hospital Germans Trias, Universitat Autònoma de Barcelona, Badalona 08916 Barcelona, Catalonia, Spain; 2 Department of Experimental Medicine. University of "Tor Vergata," Rome, Italy; 3 Lluita contra la SIDA Foundation, Institut de Recerca en Ciències de la Salut Germans Trias i Pujol,Hospital Universitari Germans Trias i Pujol, Universitat Autònoma de Barcelona, 08916 Badalona, Barcelona, Spain; 4 Statistics and Operation Research Department, Universitat Politècnica de Catalunya, Barcelona, Spain

**Keywords:** HIV, gp41, enfuvirtide, single cell death, fusogenicity

## Abstract

**Background:**

Resistance to the fusion inhibitor enfuvirtide (ENF) is achieved by changes in the gp41 subunit of the HIV envelope glycoprotein (Env). Specific ENF-associated mutational pathways correlate with immunological recovery, even after virological failure, suggesting that the acquisition of ENF resistance alters gp41 pathogenicity. To test this hypothesis, we have characterized the expression, fusion capability, induction of CD4^+ ^T cell loss and single CD4^+ ^T cell death of 48 gp41 proteins derived from three patients displaying different amino acids (N, T or I) at position 140 that developed a V38A mutation after ENF-based treatment.

**Results:**

In all cases, intra-patient comparison of Env isolated pre- or post-treatment showed comparable values of expression and fusogenic capacity. Furthermore, Env with either N or T at position 140 induced comparable losses of CD4^+ ^T-cells, irrespective of the residue present at position 38. Conversely, Env acquiring the V38A mutation in a 140I background induced a significantly reduced loss of CD4^+ ^T cells and lower single-cell death than did their baseline controls. No altered ability to induce single-cell death was observed in the other clones.

**Conclusions:**

Overall, primary gp41 proteins with both V38A and N140I changes showed a reduced ability to induce single cell death and deplete CD4^+ ^T cells, despite maintaining fusion activity. The specificity of this phenotype highlights the relevance of the genetic context to the cytopathic capacity of Env and the role of ENF-resistance mutations in modulating viral pathogenicity *in vivo*, further supporting the hypothesis that gp41 is a critical mediator of HIV pathogenesis.

## Background

HIV infection causes a progressive depletion of CD4^+ ^T cells, which leads to the development of AIDS [1,2]. Although CD4^+ ^T cell loss in HIV infection is a multifaceted process [3-5], the death of bystander CD4^+ ^T cells seems to be one of the main contributors to HIV-induced pathogenesis [6-8]. Various mechanisms have been proposed to explain the destruction of bystander CD4^+ ^T cells, including apoptosis, autophagy or abortive infection [6,8-11]. The HIV envelope (Env) glycoprotein, which mediates viral entry into the host cell by fusion of the viral and host cell membranes (reviewed in [12-14]), is one of the viral factors involved in the death of both infected [15] and bystander cells [7,8,16]. The Env complex is composed of two non-covalently linked subunits, namely, the surface glycoprotein (gp120) and the transmembrane glycoprotein (gp41), and is displayed as heterotrimers on the surface of virions and infected cells [14,17-20]. Viral entry is a multistep phenomenon: the interaction of gp120 with the host cell surface CD4-receptor, and either CCR5 or CXCR4 coreceptor enables gp41 subunits to trigger hemifusion events, thereby leading to fusion. The HIV gp41 is a classic type 1 fusion protein that contains three domains: an ectodomain, a membrane-spanning domain, and a long intracytoplasmic segment. The ectodomain of gp41 consists of an N-terminal fusion peptide followed by two conserved coiled-coil domains that are referred to as C- and N-terminal heptad repeats (HR1 and HR2), which are connected by a non-helical loop region. These HR interact with each other in a leucine zipper-like fashion to mediate membrane fusion [21]. Synthetic peptides that bind to one of the HR motifs interfere with their interaction and thus inhibit viral entry [22,23].

Enfuvirtide (ENF, T-20) is the first peptide approved for clinical use in HIV salvage therapy. This drug is a 36-amino acid peptide that was designed based on the amino-acid sequence of the HR2 domain of the gp41 subunit. This peptide prevents the HR1-HR2 interaction by binding to the HR1 domain [22,24,25]. The therapeutic benefits of ENF therapy have been demonstrated by increases in CD4^+ ^T cell counts and a significant reduction in HIV RNA levels [26-28]. Nevertheless, ENF-resistant HIV-1 variants rapidly emerge under drug pressure when virus replication is not completely suppressed [29-31]. Sequence analysis of ENF-resistant viral populations revealed the acquisition of mutations within the HR1 domain at positions 36-38 (GIV) [29,30], which were associated with a reduction in viral infectivity, probably as a consequence of impaired interaction between HR1 and HR2 [32,33]. However, certain compensatory mutations within HR2 may arise and restore viral infectivity [29,32,34-37]. Despite virological failure, specific mutations (the cluster V38A+N140I) have been associated with an increase in CD4^+ ^T cell counts [38-40].

The Env glycoprotein plays a crucial role in the depletion of CD4^+ ^T cells by inducing the death of single bystander cells, which is mediated by gp41 [41,42]. Therefore, changes in gp41 that emerge under ENF pressure could induce a change in the viral pathogenicity. Although site-directed point mutations at position 38 in gp41 have been shown to exhibit deficiency in cell-to-cell fusion activity and apoptosis induction *in vitro *and in a humanized mouse model [43,44], it is important to note that the genetic background has been proven relevant for functional evaluation of the ENF-resistant Envs because there may be compensatory changes that restore the infectivity of the virus [32,34,36,37,45,46].

The objective of the current study was to evaluate the pathogenicity of several patient-derived gp41 proteins isolated from highly experienced patients receiving an ENF-containing salvage therapy and whether changes at position 38 and 140 in gp41 have an impact in the biological properties of patient-isolated Envs. Our results indicate that the primary gp41 Env proteins, with both V38A and N140I changes, induced lower levels of single-cell death and depletion of CD4^+ ^T cells, although they retained cell-to-cell fusion activity. However, the mutation V38A in the context of a 140N or 140T change did not alter Env functions, underscoring the importance of the Env genetic background in the modulation of the cytopathic effects of the HIV-1 Env glycoproteins.

## Results and discussion

### Patients and envelope constructions

**Figure 1:**
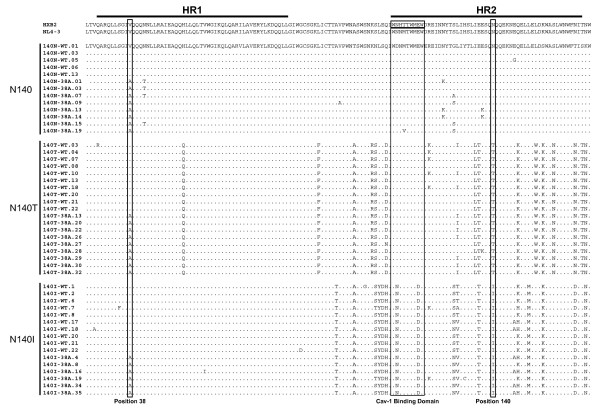
**The sequence alignment of the gp41 gene from the recombinant envelope-expressing plasmids constructed from patient samples**. The alignment of the gp41 ectodomain sequence (HR1 and HR2 domains) from the selected recombinant plasmids derived from three patients who failed an ENF-based treatment and carried a V38A mutation (HR1 box) with the amino acid I, N or T at position 140 (HR2 box). The Cav-1 binding domain in the HR2 region is highlighted.

In a previous report, we characterized gp41 proteins derived from 13 heavily pre-treated HIV-1-infected patients receiving an ENF-containing salvage therapy [29]. Several drug resistance-associated mutations were detected along the entire gp41 ectodomain, mainly mapping in the HR1 domain at positions 36, 38 and 43. Clinical findings have suggested that certain ENF-resistant mutants arising during salvage therapy, specifically, the cluster V38A+N140I, are associated with an increase in CD4^+ ^cell counts, even after virological failure [38-40,43,44]. We therefore reasoned that gp41 proteins derived from patients with different combinations of amino acids at positions 38 and 140 could have different pathogenic effects. We chose to study the gp41 proteins derived from three patients: patients 1, 9 and 10, who had mutations associated with ENF resistance at position 38 in the gp41 viral protein but differed in the amino acid found at position 140 [29]. Two plasma samples from each patient, which were collected at baseline and during treatment, were used to construct gp160 hybrid proteins (all bearing the gp120 from an NL4-3 virus and the gp41 derived from the patients). Table 1 summarizes the characteristics of the patients at the time points of viral RNA isolation. At least 15 recombinant expression plasmids were constructed from each sample, and all recombinant plasmids were fully sequenced to verify that the gp120 sequence present was conserved among the clones and that the NL4-3 wt sequence remained unchanged (data not shown). The amino acids at positions 38 and 140 of gp41 were subsequently determined, and 48 recombinant plasmids were finally selected (Figure 1). Among these plasmids, 13 clones were derived from patient 9, containing an asparagine at position 140 (140 N) and the wild-type (wt) amino acid at position 38 (38 V) or an alanine at position 38 (38A) (n = 5 and n = 8, respectively); 19 clones were derived from patient 10, who contained the substitution N140T and the wt 38 V (n = 10) or the V38A mutation (n = 9); and finally, 16 clones were derived from patient 1, who had the polymorphism N140I and the wt amino acid at position 38 (n = 10) or the V38A mutation (n = 6, Figure 1). Since we cloned the full gp41 protein present *in vivo*, we were able to identify other changes throughout the gp41 protein in addition to the changes at positions 38 and 140. Changes were found primarily in the HR2 domain, but also upstream of the HR1 domain, in the HR1 domain and in the loop section. Most of the recombinant plasmids constructed from sequences obtained during ENF treatment carried the V38A mutation as the only change associated with ENF resistance, although four clones derived from the 140N patient carried the N42T mutation, and three others showed the N126K mutation. The analysis of a caveolin-1 binding motif in the gp41 protein, which has been recently reported to affect HIV-1 pathogenesis [47], showed that only one plasmid from the patient harboring the 140N background carried an M to V substitution at position 115. The remaining plasmids constructed with sequences from this patient and all of the recombinant constructs from the other two patients showed no changes in this region before or after treatment (Figure 1). Our cloning approach, using only the gp41 of the patient instead of the entire Env (gp41 + gp120), allows us to specifically assess the effect of certain ENF resistance mutations on gp41 pathogenesis, avoiding additional effects of changes in gp120 protein itself and importantly avoiding any interference of the CXCR4 or the CCR5 coreceptor use, which is a major determinant of Env pathogenicity both *in vivo *and *in vitro *[48,49]. If viruses with different coreceptor usage had been compared, we would not have been able to discern the Env subunit responsible for the observed differences.

**Table 1:** Characteristics of the three patients receiving an enfuvirtide-containing salvage therapy when samples were collected

Patient^a^	Sample	Weeks on ENF treatment	Plasma viral load (copies/mL)	CD4^+ ^cell count (cells/μl)	No. of expression plasmids constructed
140N (9)	140N	0	366357	491	5
	V38A 140N	24	8536	700	8
N140T (10)	N140T	0	141497	13	10
	V38A N140T	4	332794	13	9
N140I (1)	N140I	0	33470	145	10
	V38A N140I	12	10806	150	6

^a^The patient IDs from a previous work [29] are indicated in parentheses.

### Cell surface expression of HIV-1 recombinant envelope glycoproteins

**Figure 2:**
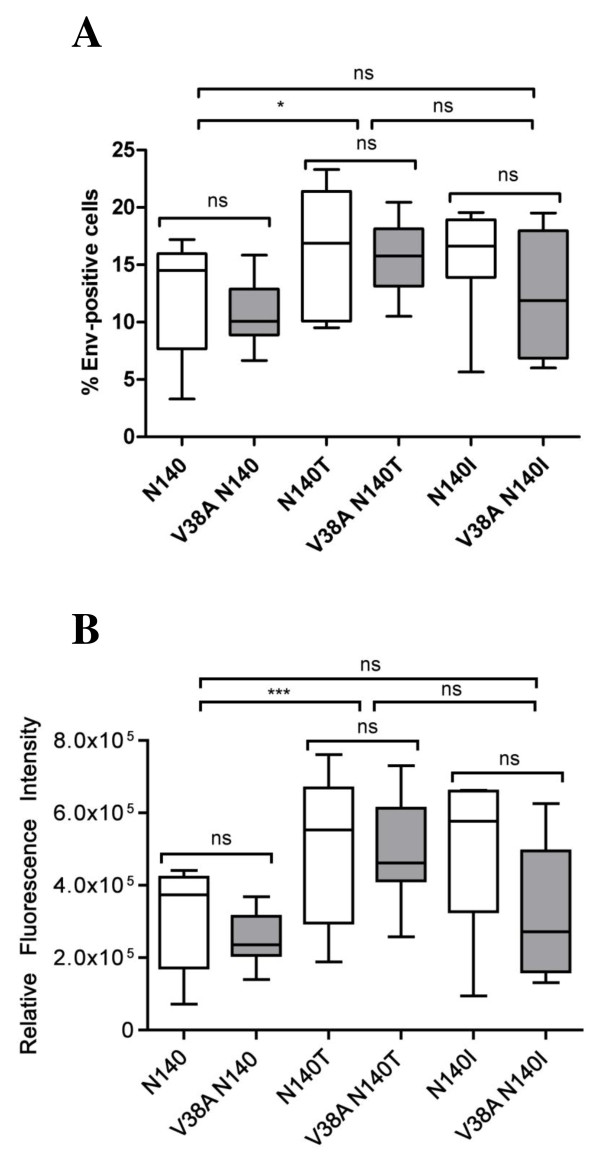
**The expression of functional recombinant envelopes on transfected HeLa cells**. Env-expressing plasmids constructed from three patients carrying an amino acid N, T or I at position 140 with (V38A) without substitutions at position 38 of gp41 were transfected into HeLa cells. The surface expression of Env was analyzed 24 hours post-transfection by staining the cells with the 2G12 antibody. Env expression was determined by the percentage of Env-positive cells in an inter-patient analysis using the clones constructed from each patient or in an intra-patient analysis using clones constructed from baseline and after treatment (V38A) samples (A). The total level of Env expression was determined by calculating the relative fluorescence intensity (RFI = % of Env-positive cells × geometric mean fluorescence of Env-positive cells) in an intra-patient analysis (B). Baseline samples (white boxes) and V38A samples (gray boxes). The boxes represent the median and interquartile range of the values. The median values were compared using a nonparametric Mann Whitney test. * p < 0.05, *** p = 0.0006, and ns denotes non-significant differences.

After the optimization of the transfection and the cell surface staining procedures (Cunyat F, Curriu M et al., *J Biomolecular Screening*, in press), HeLa cells were transiently transfected with the 48 recombinant Env-expressing plasmids, and 24 hours post-transfection, the cell surface expression of Env was analyzed using a primary antibody against the gp120 subunit (2G12). An anti-gp120 antibody was used, instead of an anti-gp41 antibody, to avoid artifacts due to changes in the gp41 protein that could affect the binding of the antibody. All tested Envs were expressed on the cell surface with expression levels ranging from 2.7% to 29.9% of positive cells (Figure 2A). When an inter-patient comparison was performed by grouping all of the Envs obtained from each patient irrespective of the time point, a significantly different percentage of Env-expressing cells was observed between plasmids constructed from the 140N- and N140T-carrying patients. The lowest percentage of Env-positive cells was observed for the 140N constructs (mean = 11.40 +/- 3.9), whereas the highest percentage of Env-expressing cells was observed for the N140T clones (mean = 15.91 +/- 4.4) (Figure 2A). However, when we analyzed the percentage of cells expressing the Env constructs obtained from the same patient by comparing clones displaying or not the V38A mutation (an intra-patient comparison after and before treatment, respectively), the Env expression was similar in all cases (Figure 2A). In addition to determining the percentage of positive cells, the Env expression levels, which could play an important role in Env pathogenesis, were evaluated. There were no differences in the Env expression levels between constructs containing wt gp41 and those containing the 38A mutation from the same patient, as determined by the geometric mean fluorescence intensity (data not shown), or the relative fluorescence intensity [50], which is a measure of the total Env expression (Figure 2B). Thus, the intra-patient comparisons suggest that the expression of Env does not change upon acquisition of the 38A mutation, and the level of Env expression is an intrinsic characteristic of the particular Env carried by each infected patient. These results allowed us to analyze the cytopathic effects of Env without correcting for cell surface expression of Env.

### Analysis of the Env protein fusogenicity

**Figure 3:**
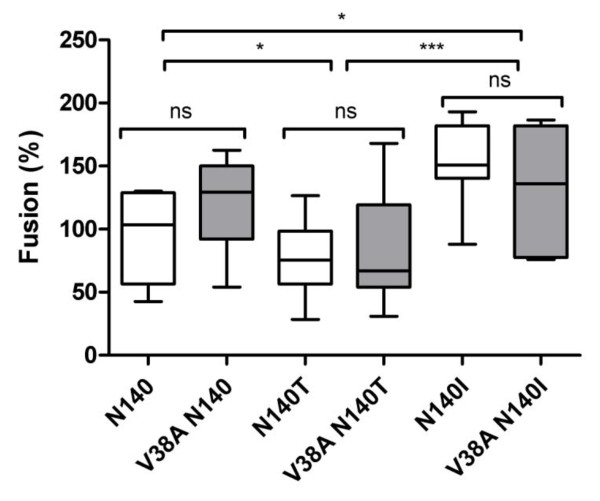
**Cell-to-cell fusion of HeLa cells expressing different patient-derived envelopes with changes at positions 38 and 140 in gp41**. Cocultures of Env^+ ^HeLa cells with TZM-bl cells were performed for 6 h, and fusion activity was determined by measuring the luciferase activity. The relative fusogenicity of each recombinant Env was calculated by normalization against the luminescence obtained using the recombinant Env of NL4-3 (100%) for each experiment. The boxes represent the median and interquartile range of the values. The median values obtained from the clones constructed from samples from each patient were compared in an inter-patient analysis, and those obtained from baseline (white boxes) and after treatment samples with the change V38A (gray boxes) clones were also compared in an intra-patient analysis using a nonparametric Mann Whitney test. * p < 0.05 *** p = 0.001 ns, non-significant differences.

The function of the HIV Env glycoprotein is to facilitate the entry of the viral nucleocapsid into the target cell. This process has an important role in HIV pathogenesis, and the fusogenic activity of HIV Env has long been associated with cytopathic effects [50,51] both *in vitro *and *in vivo *[52-54]. In agreement with this property, it has been described that single-amino-acid mutations in the ectodomain (V38A/E) or transmembrane regions of gp41 reduce cell-to-cell fusion activity of the virus [43,55]. A number of different assay systems have been reported to measure the HIV envelope activity. However, we have described the importance of selecting an appropriate cell line to express Env when the cytopathic properties of clinically derived gp41 glycoproteins *in vitro *are evaluated (Cunyat F, Curriu M et al., *J Biomolecular Screening*, in press). In this study, two envelope-expressing effector cell lines (293T and HeLa cells) were compared to evaluate the activity of patient-derived Envs: fusion, absolute cell loss and single cell death. The results showed a differential behaviour between both cell lines. 293T effector cells seem to have a rapid formation of the fusion pore, generating high levels of fusion and lower levels of single cell death. In contrast, HeLa cells would fuse slowly, inducing greater extents of single cell death. Thus, HeLa cells should be preferentially used for the evaluation of cell death parameters, and the 293T cell line should be used when envelopes with low fusogenic capacity are evaluated. Based on this recommendation, all of our recombinant Envs were first expressed in 293T cells, and assayed for fusion activity. Detectable fusion was observed for all Env, showing fusion levels over the 50% when compared with an NL4-3 wt Env (data not shown). Since all our Env were fusogenic, we used HeLa cell as the effector cell line in our assays. These cells were transiently co-transfected with the Env- and pcTat-expressing plasmids and cocultured with the reporter TZM-bl cells. After six hours of coculture, the luminescence in the sample was measured, and the relative fusion capacity of each recombinant Env was calculated in comparison to the fusion values obtained using the NL4-3 wt Env, which was used as a control (100%). The level of fusion of the Envs obtained from different patients (inter-patient analysis) showed significant differences, underscoring that the virus each patient carries may have an Env with a distinct fusogenic capacity which, in this case, is determined by gp41. Differences in fusion were not correlated with the expression level of Env on the cell surface because a higher expression level did not result in more fusion (Figures 2 and 3). However, in contrast to previously published data [43,44], when intra-patient analyses were performed, the fusogenic activities of all recombinant Envs obtained from each patient were similar, indicating that in a full-length gp41 background, the V38A mutation did not impair the cell-to-cell fusion activity of Env in the presence of the amino acids N, T or I at position 140 (Figure 3). Site-directed point mutations at position 38 in gp41 resulted in reduced cell-to-cell fusion and apoptosis induction, although viral replication *in vitro *and in humanized mice was not affected [43,44]. The differences between our data and those obtained using site-directed mutants could be explained by the fact that genetic context is extremely important in the characterization of the biological properties of Env. In the case of ENF resistance, previous studies have shown that the Env genetic background contributes to both the ENF resistance and Env function. The selection of resistance is a coevolutionary process in which HR1 mutations are selected in combination with Env variants that permit optimal phenotypic expression of HR1 mutations [45]. It is well-established that HR1 mutations introduced out of context (i.e., in a gp41 where they were not originally found) negatively impact the rate of membrane fusion; while the introduction of changes in HR2, or even in gp120, can compensate for this functional defect [29,32,34-37]. Indeed, point mutations introduced into gp41 may perturb the folding of the protein into a six-helix bundle along the HR1 and HR2 coiled-coil domains. In contrast, a native gp41 background may stabilize the fusogenic six-helix bundle with compensatory changes in the HR1 mutants, thereby minimizing the effect of the original mutation.

### Quantification of envelope-induced absolute loss of CD4^+ ^T cells

**Figure 4:**
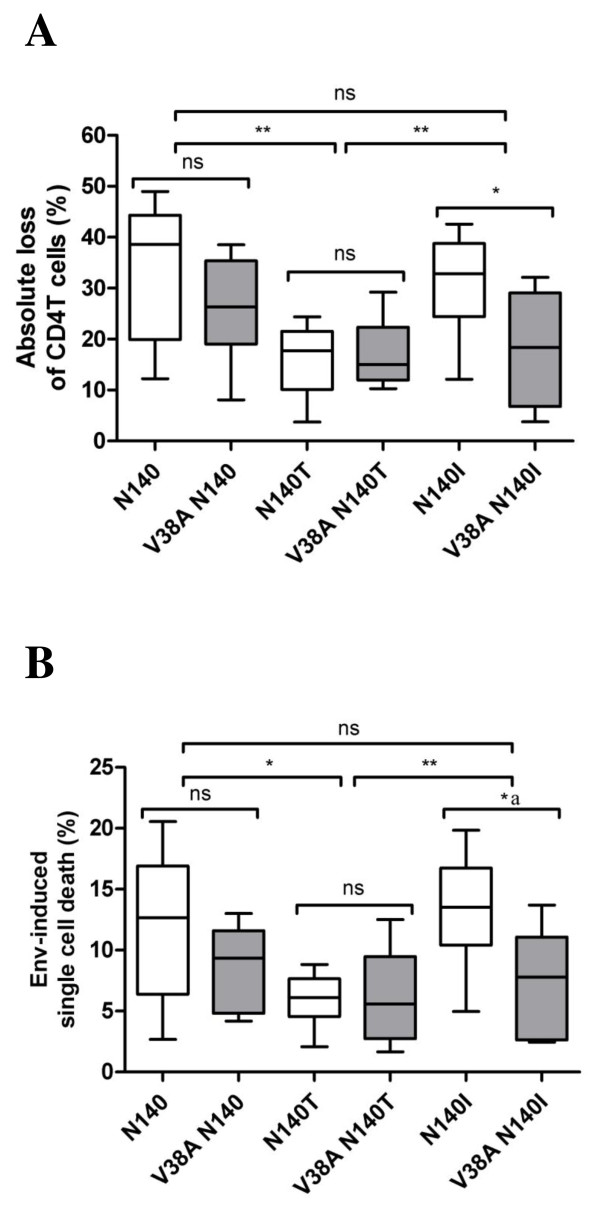
**Envelope-induced cytopathicity in CD4^+ ^T cells**. Envelope-mediated absolute CD4^+ ^T cell depletion. Absolute loss of CD4^+ ^T cells in cocultures of Env-expressing HeLa cells and purified CD4^+ ^T cells was determined. Single living CD4^+ ^T cells were morphologically gated, and the absolute loss of CD4^+ ^T cells in the coculture was quantified by the addition of PE-labeled microbeads. *p = 0.022, **p < 0.007 (A). Bystander apoptosis induced by envelopes harbouring changes at position 38 and 140 in gp41. Cocultures of primary CD4^+ ^T cells, previously stained with the DDAO cell tracker, and HeLa Env^+ ^cells were performed and analyzed after 24 hours by flow cytometry. Single, nonfused CD4^+ ^T cells were gated by the forward- vs. side-scatter characteristics and by being positives for the cell tracker. Detection of early (PI^-^DIOC^-^) and late apoptotic (PI^+^DIOC^-^) populations were determined in this gated population for each recombinant Env in the presence or absence of the coreceptor inhibitor JM-2987 (B). Boxes represent median and interquartile range of values. Median values were compared in an inter-patient and in an intra-patient assay, using a Mann-Whitney test. Baseline samples (white boxes) and V38A mutant clones (gray boxes). *^a^p = 0.031, *p = 0.0213, **p = 0.0038, and ns non-significant differences.

In addition to fusion activity, HIV Env-mediated cytopathic effects were evaluated by quantifying the absolute loss of purified primary CD4^+ ^T cells cocultured with HeLa cells that expressed the recombinant Envs. Despite a similar fusogenic capacity of the wt recombinant Envs and those bearing mutations at position 38, the absolute loss of CD4^+ ^T cells was significantly lower after exposure to Envs containing the V38A mutation in a N140I background than after exposure to wt Env (18.1% and 31.3%, respectively. P = 0.022) (Figure 4A). In contrast, Env proteins containing either an N or a T at position 140 induced comparable levels of absolute CD4^+ ^T cell loss, irrespective of the amino acid present at position 38 (33.4% 38V and 25.6% 38A in an 140N background and 15.8% and 17.0%, respectively, with a 140T change, Figure 4A).

### Analysis of envelope-induced bystander apoptosis

The Env-mediated depletion of the CD4^+ ^T cells may result from syncytium formation (directly related to fusogenicity values) or from gp41-mediated bystander apoptosis of single CD4^+ ^T cells. Apoptosis is a crucial factor contributing to the loss of CD4^+ ^T cells, and the extent of immune-cell apoptosis is correlated with AIDS progression [56]. Importantly, apoptosis of bystander, uninfected cells is one of the major processes involved in the destruction of immune cells during HIV infection [8] because the majority of apoptotic CD4^+ ^T cells in the peripheral blood and lymph nodes are uninfected in patients infected with HIV [57,58]. The Env-induced death of single bystander cells is related to gp41-mediated membrane hemifusion processes between Env-expressing cells and target cells [41,42]. This gp41 dependence suggests that drugs targeting gp41 function may alter HIV pathogenesis by inducing changes in the gp41 sequence. Consistent with this, certain ENF-resistant mutations arising during salvage therapy, specifically changes at position 38 in gp41, were associated with an immunological benefit, even after virological failure [39,40]. Additionally, the increase in CD4^+ ^T cells was enhanced by the concomitant polymorphism N140I [38]. Because there were no significant differences in fusogenicity among the Envs tested in our assays, we specifically quantified Env-mediated cell death, since *in vitro *the single-amino-acid mutation V38A/E has been shown to alter this mechanism [43]. HeLa cells expressing recombinant Envs were cocultured with primary CD4^+ ^T cells. After 24 hours, the culture was stained with propidium iodide (PI) and DiOC_6_(3) to simultaneously determine the viability and the mitochondrial transmembrane potential of CD4^+ ^T cells, respectively. Each Env was analysed in the absence and presence of the anti-CXCR4 antagonist JM-2987, and a correction for non-Env-mediated death was performed for each Env by subtracting the background death detected in the presence of JM-2987. When an intra-patient analysis of this assay was performed, there were differences between the recombinant clones carrying the V38A mutation and the wt clones in an N140I background, while the clones bearing the N or T amino acids at position 140 showed similar apoptosis-inducing capacity when wt and V38A clones were compared (Figure 4B). Thus, the impaired ability to deplete CD4^+ ^T cells by the recombinant Envs that contained the cluster of mutations V38A+N140I in the gp41 protein was associated with a significant reduction in apoptosis induction on primary cells (13.4% and 7.4% for baseline and with the cluster of mutation samples, respectively; p = 0.031). In agreement with previously reported *in vivo *data [38], both the absolute loss of CD4^+ ^T cells and Env-induced single cell death was significantly reduced for Envs that had the cluster of mutations V38A + N140I. However, the acquisition of the V38A mutation in a gp41 background containing an N or T at position 140 had no significant impact on the pathogenicity of Env, again highlighting the importance of the genetic context on the function of Env. Polymorphisms in HR2 have been shown to contribute to ENF resistance [45], and in this study, we demonstrate that they also have an important role in HIV pathogenesis. Nevertheless, a methodological limitation of our study is the relative small number of clones analyzed which gives us a low statistical power. A larger sample size would allow a more accurate estimation of the differences studied.

Recently, it has been reported that caveolin-1 modulates the Env-induced bystander apoptosis through interactions with gp41. None of the clones obtained from the patient carrying the N140I mutation in gp41 showed changes in the caveolin binding region, suggesting that the changes in the induction of bystander apoptosis observed in this study are not due to a defect in the binding of this protein to the gp41 protein.

## Conclusions

Overall, the phenotype observed in the present study for Envs containing the cluster of mutations V38A+N140I, which maintained fusion capacity, but had a decreased ability to deplete CD4^+ ^T cells, correlated with the observed *in vivo *data in patients, including a maintenance of viral load with an increase in CD4 counts. These results show that mutations that confer ENF resistance are associated with reduced pathogenicity *in vivo*.

In conclusion, these findings support the hypothesis that HIV gp41 is a critical mediator of HIV pathogenesis and suggest that it may be possible to target gp41 to attenuate HIV.

## Methods

### Patients

Three highly-experienced patients who were receiving an ENF-containing salvage therapy were selected from our previous study [29]. These patients carried viruses that developed mutations at position 38 in the gp41 viral protein associated with drug resistance and had different changes at position 140. Two plasma samples from each patient were collected at baseline and during ENF treatment and were used to extract viral RNA.

### Cell cultures and reagents

HeLa and TZM-bl cell lines were supplied by the NIH AIDS Research and Reference Program. The cell lines were grown in Dulbecco's modified Eagle's medium (DMEM) supplemented with 10% of heat-inactivated fetal calf serum (FCS) and maintained at 37°C in a 5% CO_2 _incubator. Peripheral blood mononuclear cells (PBMCs) were freshly isolated from buffy coats obtained from a local blood bank (Banc de Sang i Teixits, BST), and CD4^+ ^T cells were purified by negative immunomagnetic selection (Miltenyi Biotec, Spain). The final cell preparations were composed of > 95% CD4^+ ^T cells as determined by flow cytometry. The isolated CD4^+ ^T cells were incubated overnight at 37°C in RPMI media supplemented with 10% of FCS prior to use. All of the media were purchased from Invitrogen (Madrid, Spain).

The CXCR4 antagonist JM-2987 (hydrobromide salt of AMD-3100) [59] and the CCR5 antagonist TAK-779 [60,61] were obtained through the NIH AIDS Research and Reference Program. The broadly gp120 neutralizing antibody 2G12 and the secondary antibody goat anti-Human IgG were obtained from Polymun (Vienna, Austria) and Jackson ImmunoResearch Laboratories (Pennsylvania, USA), respectively. The Tat expression plasmid pcTat was obtained through the NIH AIDS Research and Reference Reagent Program [62].

The cell tracker Dichloro-DimethylAcridin-One (DDAO) was purchased from Molecular Probes (Invitrogen, Madrid, Spain). The cationic fluorescent dye Propidium Iodide (PI) and the potentiometric mitochondrial probe DIOC_6_(3) were purchased from Sigma (Madrid, Spain) and Invitrogen, respectively.

### Plasmid construction

The RNA from the plasma samples was isolated before and after the initiation of ENF treatment using the QIAmp Viral RNA kit (Qiagen). Full-length env/rev genes were amplified through RT-PCR using specific primers as previously described [63]. A subsequent nested PCR was carried out using Platinum^® ^Taq DNA Polymerase High Fidelity (Invitrogen) to obtain a fragment corresponding to the gp41 protein (primers MluF2 and RNANestedR corresponding to nucleotides 7726-7747 and 8882-8904 of the HIV _HXB2 _numbering system, respectively). A fragment corresponding to the gp120 protein was amplified from a NL4-3 plasmid (primers RNANestedF and MluR2 corresponding to nucleotides 5954-5983 and 7727-7747 of the HIV _HXB2 _numbering system, respectively). The purified gp41 and gp120 products, which overlapped each other in 22 bases, were combined by PCR and purified to obtain the recombinant Envs (gp120 from NL4-3 and gp41 from patients). A directional cloning reaction was performed to insert the fragment into the plasmid expression vector pcDNA.3.1D/V5/His-TOPO (Invitrogen), and several transformed bacterial colonies were selected for each sample. All recombinant plasmids were sequenced using specific primers, the Big Dye Terminator v3.1 cycle sequencing kit (Applied Biosystems) and an automatic DNA Sequencer (3100 Genetic Analyzer). The sequences were edited (using Sequencher, v4.7, from the Gene Codes Corporation, Ann Arbor, MI and GeneDoc, v2.6, software), and the recombinant plasmids with the required mutations were selected.

### Transfections

HeLa cells were plated at a density of 8 × 105 cells/well in six-well plates and allowed to grow overnight. The cells were transiently transfected (using Lipofectamine 2000 Reagent, Invitrogen, Spain) with 1.3 μg of the Env-expressing plasmids for the cocultures with primary cells or were cotransfected with the Env-expressing plasmids and 2.7 μg of pcTat for the fusion assays. Twenty-four hours post-transfection, the cells were collected for further analyses. As negative controls, cells were mock-transfected (with the pcDNA 3.1 vector) or transfected with pcTat alone.

### Envelope expression

Twenty-four hours post-transfection, cell membrane expression of the Env glycoprotein was assessed by flow cytometry after indirect staining with the anti-gp120 monoclonal antibody 2G12 (4 μg/ml) for 20 min at 37°C, followed by staining with phycoerythrin-labeled goat anti-human IgG (RT for 15 min). The cells were washed, fixed in 1% formaldehyde and analyzed by a FACS LSRII flow cytometer. The data were analyzed using FACSDiva software (BD Biosciences). Mock-transfected cells were used as a negative staining control. The percentage of Env-positive cells and the geometric mean fluorescence intensity (geoMFI) of these cells were considered as individual parameters or used to calculate the relative fluorescence intensity (RFI = % of Env-positive cells × geoMFI of Env-positive cells), as described previously [50].

### Cell-to-Cell Fusion assays

Twenty-four hours post-transfection, Env/pcTat- and pcTat-transfected HeLa cells were cocultured with the reporter cell line CD4^+^/CCR5^+^/CXCR4^+ ^TZM-bl for 6 hours in 96-well plates in the presence or absence of the CXCR4 and CCR5 co-receptor inhibitors JM-2987 and TAK-779 (1 μg/ml), respectively. The fusion efficiency of each clone was quantified by assessing the luminescence of the cells (Britelite kit, Perkin Elmer) with a Luminoskan Ascent luminometer (Labsystems, Spain).

### Envelope-induced death in primary CD4^+ ^T cells: absolute cell loss and bystander apoptosis

Env-induced cytopathic effects were evaluated using a coculture system of Env-expressing HeLa cells as effector cells and labeled primary CD4^+ ^T cells as target cells. The primary CD4^+ ^T cells were stained with the far red cell tracker, DDAO (10 μg/mL), for 1 hour at 37°C. Env^+ ^HeLa cells and CD4^+^/DDAO^+ ^T cells were cocultured for 24 hours in the absence and presence of the inhibitor, JM-2987 (1 μg/mL), and were stained with DiOC_6_(3) (40 nM) and PI (5 μg/mL) for 1 hour at 37°C. Labeled microbeads (Beads Perfect Count, Invitrogen) were added to the stained coculture to quantify the absolute cell loss, and flow cytometry was performed by a FACS LSRII flow cytometer. The data were analyzed by the FACSDiva software (BD Biosciences).

### Statistical analyses

The data were compared using non-parametric Mann-Whitney tests. All statistical analyses were performed using GraphPad Prism, version 5.01, for Windows (GraphPad Software, San Diego, California, USA). A P-value of 0.05 was considered to be significant for these studies.

## Competing interests

The authors declare that they have no competing interests.

## Authors' contributions

FC, JB and CC together designed this study. FC, EG and MC performed the plasmid constructions, the fusogenicity and the cell depletion assays. FC and N P-A performed the statistical analysis. FC, VS, CP, JB and CC drafted and edited this manuscript. SM was responsible for sequencing the Envs. All authors have read and approved the final manuscript.
